# Mental Images and School Learning: A Longitudinal Study on Children

**DOI:** 10.3389/fpsyg.2019.02034

**Published:** 2019-09-18

**Authors:** Maria Guarnera, Monica Pellerone, Elena Commodari, Giusy D. Valenti, Stefania L. Buccheri

**Affiliations:** ^1^Faculty of Human and Social Sciences, Kore University of Enna, Enna, Italy; ^2^Department of Educational Sciences, University of Catania, Catania, Italy

**Keywords:** children, mental imagery, school, learning, cognitive process

## Abstract

Recent literature have underlined the connections between children’s reading skills and capacity to create and use mental representations or mental images; furthermore data highlighted the involvement of visuospatial abilities both during math learning and during subsequent developmental phases in performing math tasks. The present research adopted a longitudinal design to assess whether the processes of mental imagery in preschoolers (ages 4–5 years) are predictive of mathematics skills, writing and reading, in the early years of primary school (ages 6–7 years). The research lasted for two school years; in the first phase, the general group of participants consisted of 100 children, and although all participants agreed to be part of the research, in the second phase, there was a mortality rate of 30%. In order to measure school learning and mental imagery processes four batteries of tests were used. The mental imagery battery evaluated mental generation, inspection and transformation processes. Data underlined that the different aspects in which mental imagery processes are articulated are differently implied in some skills that constitute school learning. These findings emphasize the potential usefulness of a screening for mental imagery ability for schoolchildren to adopt effective measures to increase their mental imagery abilities.

## Introduction

The mental image or mental representation is defined as what an individual can feel, hear, see or taste in one’s own mind. However, the stimulus which creates the image is not actually seen, heard, felt, tasted, or smelt (Fennema, 1959; Richardson, 1969; Coon and Mitterer, 2011).

Most cognitive processes, for example perception, memory and imagery, involve mental representations or mental images (Pellerone et al., 2017; Guarnera et al., 2018). In processes of perception, each stimulus is interpreted and integrated into a mental image; memory and imagery allow for the generation and manipulation of images of objects or scenes without sensory stimuli.

Therefore, from a generic point of view, mental imagery can be considered as a cognitive process that represents reality through multisensory mental images, or mental representations of perceived or remembered objects. From a specific point of view, it is the ability to manage mental images in order to perform a task (Richardson, 1969; Kosslyn, 1994; Coon and Mitterer, 2011).

Kosslyn (1999) described different mental imagery processes - such as generation, inspection, maintenance, and transformation.

Pearson et al. (2013) provide a clear definition of these different processes. Generation is the capacity to create mental representations without a perceived stimulus. In particular, an image can be created directly from immediate perceptual information, (for example, someone can look at a picture of an object, create a mental image in their mind, and then maintain this mental image as they look away or close their eyes) or created entirely from previously stored information held in long-term memory.

Maintenance describes the capacity to maintain images in the short-term memory. In fact, once generated, a mental image is subject to rapid decay with an average duration of only 250 ms, which corresponds to the time necessary to make an eye movement (Kosslyn, 1994). This brief duration means that active maintenance of the image is required to perform other imagery processes (inspection or transformation).

Inspection is the capacity to explore a generated image in order to interpret an object-based characteristic or spatial property of the image. For example, to describe the shape formed by a fox’s ear a person would generate a mental image of a fox and then examine the shape of the ears within the image in order to produce a response (Kosslyn et al., 2001).

Transformation is the capacity to modify mental image. Two of the most extensively researched types of image transformation are that of mental rotation, the ability to rotate mental representations of two-dimensional and three-dimensional objects, and image restructuring, in which the interpretation of a mental image is changed or modified in some way.

Literature have underlined the connection between children’s reading skills and the capacity to create and use mental representations (Ehri and Wilce, 1982; Share, 1999; Apel and Masterson, 2001; Bara et al., 2004; Pellerone, 2013). Reading skills are characterized by the capacity of acquiring orthographic and phonological representations and being be able to create connections between them (Ehri, 1995; Metsala and Ehri, 1998). The relationship between the development of learning, such as reading and writing, and the capacity to form mental images of viewed words has been also measured by researches conducted on subjects with dysgraphia and dyslexia. Koenig et al. (1991) discovered that individuals with dyslexia performed worse than those without dyslexia on mental generation tasks, only when the stimuli were letters, but not when diverse stimuli were used. According to the authors the difficulties which people with dyslexia faced could be related to problems in the integration process of the segments which compose letters in a mental image. Examined the mental rotation ability in children with developmental dyslexia aged 7–9 years. They used tasks including three-dimensional figures, mental rotation of letters, and colored pictures of humans or animals, founding that individuals with dyslexia tend to be impaired in mental rotation for all three stimuli; furthermore, subjects with dysgraphia seem to manifest similar difficulties. In particular, a study conducted on individuals with dysgraphia showed that the errors in writing tasks may be linked to a buffer graphemic deficit (Yachini and Friedmann, 2010). Roncoli and Masterson (2016) analyzed a 10-year-old child with spelling difficulties but good reading abilities, showing that the child’s performance on a 60-word (45 regular and 15 irregular) dictated task was lower than that of a group of peers. The child’s visual-memory performances, verbal short-term memory, rapid naming, and phonological awareness, were similar to those of the comparison group, but the child’s difficulties were related to mental representations of letters; in fact, the child was usually capable to report the first three letters of arrays.

Visual-spatial representations are used extensively in mathematics (Kirby et al., 1988; Hegarty and Kozhevnikov, 1999; Kozhevnikov et al., 2005; van Garderen, 2006; Blazhenkova, 2016). Moreover spatial ability, generically intended as the ability to mentally represent and transform two-dimensional and three-dimensional images (Wang and Carr, 2014), is involved in the math learning (Hegarty and Kozhevnikov, 1999; McKenzie et al., 2003; Rasmussen and Bisanz, 2005; van Garderen, 2006; Barnes et al., 2011), and during subsequent developmental phases (Battista, 1990; Casey et al., 1995; Reuhkala, 2001).

Hegarty and Kozhevnikov (1999) underlined that, during the learning of mathematics, children tend to use two types of visuospatial representations: schematic representations and pictorial representations. Schematic representations encode the spatial relations described in a math problem; pictorial representations encode the visual appearance of the objects described in the problem. Schematic representations are connected to better performance on arithmetic problems.

Several studies (Levine et al., 1985; Farah et al., 1988; Milner and Goodale, 1995; Kosslyn et al., 2001; Kozhevnikov et al., 2005; Kozhevnikov and Blazhenkova, 2013) distinguished between object and spatial-visual imagery. Object imagery refers to representations of the literal appearances of individual objects and scenes in terms of their shape, color, and texture. Spatial imagery refers to representations of the spatial relations among objects, locations of objects in space, movements of objects and their parts, and other complex spatial transformations.

McKenzie et al. (2003) showed the presence of a higher number of errors in arithmetic tasks among children aged 6–7 year-old, compared to older children, in a condition of visuospatial interference with respect to those in a verbal-interference condition.

Also, a recent longitudinal study investigated the developmental trajectories of the predictive relationship between spatial ability and early numerical skills in elementary school children as they progressed from the second to fourth grade (Carr et al., 2018). The authors showed that spatial skills development presents two developmental profiles predicted by socioeconomic status, verbal working memory, and gender; in turn the developmental profiles predicted differences in mathematics achievement.

Research has widely documented the link between mental imagery and school learning. Several studies have investigated the link between mental imagery processes and the specific abilities involved in academic learning among preschoolers. Moreover, many researches showed the effect of preschool children’s abilities on the formal learning of reading, writing and math both early schooling and in the following school grades. These early skills appear to consist of several nuclear factors or learning skills precursors, such as phonological awareness, alphabet knowledge, notational knowledge, textual knowledge respect to reading and writing, understanding of number concepts respect to math (for example Evans et al., 2000; Vellutino et al., 2004; Pinto et al., 2008; Jordan et al., 2009).

However the different factors take on a different weight in relation to the different stages of acquisition of formal learning (for example La Paro and Pianta, 2000; Orsolini et al., 2006; Pinto et al., 2009, 2012; Gunderson et al., 2012).

Moreover several domain-general factors, such as attentional, perceptive, memory, motor and linguistic processes (and their sub-processes), have also been found to contribute to both learning skills precursors and subsequent scholastic competences (for example, D’Amico and Guarnera, 2005; McCutchen, 2005; Ecalle et al., 2008; Commodari and Di Blasi, 2014; Guarnera and D’Amico, 2014; Carr et al., 2018).

Among the different domain-general factors mentioned above, even mental imagery processes are included.

In reference to reading and writing skills precursors, it is known that the acquisition of notational knowledge (which includes among the various aspects the child sensitivity to signs in relation to sounds in the written code and to constraints on how the letters in written words are organized), is favored by visual coding. Visual coding, involves mental imagery in as much concerns the ability to encode, store, and retrieve visual information, involving sensory and higher-level visualization processes that are implied in the storage of representation defining the visual attributes of environmental stimuli, such as the graphic symbols used to represent written words (Vellutino et al., 2004; Pinto et al., 2009, 2012; Commodari et al., 2019). Therefore children who have a good conceptual knowledge on orthography have available in their memory the orthographic representation of the letters of a word. This factor in preschool children is a crucial ability because it allows correct coding and decoding of written signs and is highly related to their competences in all early writing and reading tasks (Pinto et al., 2012).

As regards the math skills precursors it is known that spatial ability (as mental transformation ability), can improve prescholar children’s development of numerical knowledge by helping them to create a linear spatial representation of numbers. In turn, a strong linear number representation improves other aspects of numerical knowledge, such as arithmetic estimation. One theory that explains how spatial skills may support mathematics achievement (Lemer et al., 2003; Feigenson et al., 2004) assumes that two core systems of the number are grounded in separate neural networks. One is approximate and non-symbolic and it is assumed that shares neural codes with spatial skills. The other is precise and symbolic and primarily is recruited by exact counting and symbolic math operations (Feigenson et al., 2004). Developmentally, as the two core systems of number merge through schooling, spatial skills will increasingly impact mathematics achievement also via the second symbolics system of number (Piazza et al., 2013).

Other researches that have studied the relation between mental imagery and the preschoolers’ abilities linked to school learning show that preschool children rapidly create mental images of letters and words with reduced exposure (Ehri and Wilce, 1982; Jorm and Share, 1983; Apel and Masterson, 2001; Ehri, 2005; Apel et al., 2006). For example, Apel et al. (2006) have shown that preschool children (5-year-old) can rapidly acquire the mental orthographic images of new words. The authors visually showed some non-words to the children, accompanied by the figure of a new object representing the non-words. According to the results, the preschool children, by integrating auditory and visual information, were able to form mental representations of the spellings of words. Bara et al. (2004) underlined that: the task of creating a link between the orthographic representations and the corresponding phonological representations of words can be considered as one of the difficulties in read learning process.

Literature underlined as multisensory learning programs help preschoolers children form links between orthographic representations and the corresponding phonological representations of words by touch; in particular, Bara et al. (2007), in a study on subjects aged 5–6 years discovered that, after visual-haptic training, the recognition abilities of letters and reading pseudo-words was better compared to a subjects who had only attended a visual training.

Guarnera et al. (2017), in a study conducted on children aged 4 and 5 years, found that the capacity to generate tactile and visual mental images of previously perceived stimuli and the capacity to generate and inspect mental images (without an external object) influence the acquisition of school readiness skills, such as logical-mathematical, phonological and linguistic abilities. In a longitudinal study (children aged 6–10), Zhang et al. (2014), to measure the spatial visualization, that is a spatial ability involving multi step manipulations of spatial information (Linn and Petersen, 1985), administered a spatial-relations task to a group of preschoolers. The results of the study showed that children with a stronger spatial visualization ability in pre-school showed higher arithmetic abilities when starting elementary school and later showed a faster rate of growth in arithmetic.

## Research Aim

Although the link between mental imagery and school learning is well documented, previous studies have analyzed only some aspects of mental imagery involved in academic learning, focusing their attention only on some learning areas (Zhang et al., 2014). With the aim to overcome these limitations, the present study - adopting a longitudinal design, based on Kosslyn (1999) - assessed how the different processes of mental imagery - such as generation, inspection and transformation - are predictive of reading, writing, and mathematics ability, in the early years of primary school.

In particular, the present research analyzed whether the diverse aspects of processing of mental imagery (such as generation, inspection and transformation processes), during two different developmental stages (4–5 years versus 6–7 years old), are equally implicated in all abilities that constitute school learning or only in a part of these.

## Materials and Methods

### Participants

The group is formed by 100 preschoolers, of which 50 girls and 50 boys aged between 4 and 5 years old (*M* = 4.5; *SD* = 0.50), who attended two preschools in a large Italian town. In a subsequent phase of the study, 2 years later, only 70 of the 100 children who participated in the first phase of the study were traced (47% boys and 53% girls with mean age of 6.5, *SD* = 0.50, range = 6–7 years), with a mortality rate of 30%. The research lasted for two school years, conducted between 2016 and 2018.

Inclusion criteria included the desire and satisfaction to participate in the research; exclusion criteria included having a mental and/or physical disability, certified by Italian Public Health System. To check the inclusion and exclusion criteria, a semi-structural interview was conducted with participants and their parents.

The Internal Review Board (IRB) of Faculty of Human and Social Sciences at the University of Enna “Kore” has approved the administered instruments and the research project.

The tests were administered individually. All study participants were given information of the study; furthermore, the informed written consent was signed from the parents of the participants in this study, where voluntary participation, guarantee of anonymity, free will of withdrawal from the participation, and no disadvantage upon withdrawal were explained. Upon written consents from the parents’ subjects, data was collected. The instruments were administered by qualified researchers, and children were given 40–45 min to complete them.

The participants are from a medium to high socioeconomic background.

### Materials

In order to measure academic learning and mental imagery processes four batteries of tests were used.

Academic learning was evaluated. Reading proficiency was tested with the standardized MT Battery of Italian reading, which assesses reading comprehension, accuracy (or correctness), and speed (or fluency) using materials that corresponded to those the child would typically read in school (Cornoldi et al., 1995). The comprehension test measures the understanding of a written text. The task consists of a written text suitable for the sample’s age and ten multiple-choice questions formed using words different from those in the text. The total number of correct answers is scored. The accuracy and speed tests require participants to read a text printed on a card with the instruction to read aloud as fast as possible and to avoid mistakes, respectively. The examiner had a schedule to evaluate performance. The task has no time limit, but a subject’s time is recorded. The accuracy score is calculated by summing the mistakes made during the reading of the text, whereas the speed score is the number of seconds spent reading the text divided by the number of syllables in the text. Therefore, from both the accuracy and speed tests, one can infer that lower scores represent better performance. The Cronbach’s alpha coefficient is from 0.70 to 0.77 for the Comprehension test; from 0.75 to 0.89 for the Accuracy test, and from 0.94 to 0.96 for the Speed test.

Writing skill was evaluated through two tasks taken from a standardized Italian battery (Tressoldi and Cornoldi, 2000): dictation and spontaneous writing. The first test involves the repetition of a piece that is age appropriate for the subjects; for each mistake, 1 point is assigned. For the Spontaneous Writing test, the child must describe a figure by writing a story; the time available is 10 min and each spelling mistake is counted. From both the Dictation and Spontaneous Writing tests, one can infer that lower scores represent better performance. The Cronbach’s alpha coefficient is equal to 0.68 for the Dictation Writing test and 0.72 for the Spontaneous Writing test.

Arithmetic abilities were assessed using the AC-MT standardized mathematics test (Cornoldi et al., 2002). This test evaluates calculation competence using tasks divided according to scholastic grade; it is divided into two parts. The first part consists of five tasks that can be administered individually or collectively. The first task, written calculation, measures a child’s ability to solve basic operations. The second task, size discrimination, measures a child’s ability to discriminate the size of a number. The third task, word–number transcoding, evaluates lexical and syntactic numerical knowledge. The fourth and fifth tasks, number ordering tasks, assess semantic and syntactic knowledge. In the second part of the test, the time taken to perform the tasks is recorded. This part of the battery includes mental calculation, written calculation, enumeration, and numerical facts tasks. This last task assesses basic knowledge of addition and subtraction with one-digit numbers and other facts. A child’s response is considered correct only if it is given immediately and not as a result of a computation. AC MT 6–11 (Cornoldi et al., 2002) supplies four global scores: written calculation, numerical knowledge, calculation accuracy, and calculation speed. Scores reflect the main components of calculation competence. Written calculation score refers to written calculation ability. The score is the number of correct responses in the written calculation task. The Cronbach’s alpha coefficient is equal to 0.84; the reliability for each task is satisfactory, in particular: for Written Calculation the Cronbach’s alpha coefficient is equal to 0.74, for Numerical Knowledge is equal to 0.68, for calculation accuracy is 0.77, and for calculation speed is 0.63.

Numerical knowledge includes the set of abilities fundamental to a child’s understanding of the concept of numerical quantity and its modifications. The score is the number of correct responses obtained in the size discrimination, word–number transcoding, and number ordering tasks. Calculation accuracy score reflects a child’s ability to correctly process information during a calculation. The score is the number of errors in the second part of the battery.

Calculation speed score is a measure of calculation speed. It reflects the automation of the calculation process. The score is obtained from the sum of the response times in the written calculation, mental calculation, and enumeration tasks. Therefore, from both accuracy and speed tests, one can infer that lower scores represent better performance.

In order to measure visual imagery processes, an *ad hoc* battery of tasks was used. The battery consists of three tasks, such as *Blind touch, Are letters and forms similar?*, and *Snail’s walk*, and it measures different aspects of the mental imagery process.

The *Blind touch* (Cronbach’s α = 0.65), measures generation process. The task presents 5 forms, which are snail, tire, paintbrush, moon and home. The subjects are blindfolded, and the forms are hidden in a bag. The recognition and naming of the forms which are extracted randomly in done trough tactile exploration. As the battery tasks are administered in diverse orders, if this task is first, the children are given several minutes to become familiarized with the objects. Familiarization consists in exploring them by touching and observing them freely. The test takes 5–10 min, and each correct answer is assigned 1 point.

The *Blind touch* task, consisting in the recognition of the forms through the tactile sensory modality, involves the generation of a multisensory image. In fact, during the exploration phase, a mental multisensory representation of the object seen and touched is created directly from immediate perceptual information. Subsequently, during exclusively tactile stimulation, in order to perform the task, a link between the actual tactile representation of the external stimulus and the visual and tactile mental image of the previously perceived object must be made.

The second which is called one, *Are letters and forms similar?* (Cronbach’s α = 0.76), measures inspection process. The task consists of the same objects used in the first task (home, moon, paintbrush, tire, and snail) but these resemble 5 letters (A, C, I, O, and P), which are printed in black on a white paperboard. They are shown to subjects for a few minutes to allow them to become familiar with them. After this, the letters are hidden and substituted by the forms, which are presented one by one and the same order is never changed. The instructions are to associate each object with its corresponding letter. The task lasts around 5–7 min, 0 points is assigned for incorrect answers and 1 point for correct answers.

The *Are letters and forms similar?* task consists in recognizing the similarity between two objects, through the association of the forms and corresponding letters. It concerns the capacity to generate and inspect a mental image, such as the mental representation of a previously perceived external stimulus (letter), in order to detect the characteristics that resemble the currently perceived external stimulus (form).

The third one, which is called *Snail’s walk* (Cronbach’s α = 0.85), measures transformation process. The task presents an *L*-shaped path on a sheet of A4 paper, which has four diverse colored circles (blue, green, red, and yellow) on each side, and the snail form used in the previous tasks. The children must try to mentally represent the snail’s route from one circle to another. They have to establish which circle the snail’s shell is turned to. Questions with possible directions, 4 answers in total, were proposed to the children, and 1 point is assigned for each right answer. The task lasts about 10–15 min.

The *Snail’swalk* task consists in identifying the orientation of a part of an object through the mental representation of the movement of the object along a path in space. This task requires the ability to generate the image of the previously perceived path and generate and transform the image of the object (the snail), rotating it to determine the orientation of a part of it (the shell).

These mental imagery tasks require the activation of one or more mental imagery processes;, in particular, the cognitive effort required by a task (in terms of working-memory involvement) is changed by the type of mental imagery process activated (Hussey et al., 2012; Di Nuovo et al., 2014; Castellano et al., 2015).

## Data Analysis

All the analyses were conducted with Statistical Package for the Social Sciences 23.0 (IBM Corporation, Armonk, NY, United States).

The descriptive analysis was used to assess the mean scores of all variables at Time 1 and Time 2.

In reference to the Time 1, the Univariate Analysis of Variance (ANOVA one-way) was used to measure the influence of age on visual imagery processes, measured with *Blind touch, Are letter and form similar?*, and *Snail’s walk.*

In reference to the Time 2, the Multivariate Analysis of Variance between-subjects design (MANOVA) was carried out to inspect the potential effect between the age variable and visual imagery processes on academic abilities.

Furthermore, the partial correlation was carried out to verify the role of independent variables in order to value their effect on dependent variable by eliminating the age variable.

Separate hierarchical regression analyses for each dependent variable (reading: comprehension, accuracy, and speed; writing: dictation and spontaneous writing; calculation: written calculation, number knowledge, accuracy, and speed) were conducted to evaluate the contribution of the different mental imagery processes on school learning during two different developmental stages (ages 4–5 and ages 6–7). A hierarchical regression is the general approach of estimating the regression equation by considering a defined set of variables. This analysis allows to predict or explain scores on a criterion variable on the basis of obtained scores on predictor variables and knowledge of the relationships among all the variables.

## Results

The descriptive analyses for all variables (school learning and mental imagery) are reported in Table 1.

**TABLE 1:** Descriptive statistics at Time 1 and Time 2.

**Variables**	**Min**	**Max**	***M***	***SD***
Blind touch at Time 1	0	5	4.27	1.03
Are letters and forms similar? at Time 1	0	5	3.41	1.66
*Snail’s walk* at Time 1	0	4	2.30	1.60
Blind touch at Time 2	2	5	4.41	0.84
Are letters and forms similar? at Time 2	2	5	4.69	0.63
*Snail’s walk* at Time 2	0	4	2.79	1.05
Reading: comprehension	3	15	8.57	2.31
Reading: accuracy	0	12	4.09	2.95
Reading: speed	0	1	0.83	0.38
Writing: dictation	0	20	5.10	4.09
Writing: spontaneous writing	0	150	25.69	25.74
Calculation: written operation	0	4	2.71	1.43
Calculation: number knowledge	0	26	15.70	7.33
Calculation: accuracy	0	20	6.51	5.10
Calculation: time	29	178	86.79	28.88

*The increase of the scores in dictation, spontaneous writing, calculation accuracy, and time indicates a deterioration of the performance (see section “Materials”). Min, minimum; Max, maximum; M, media; SD, standard deviation.*

In reference to the Time 1, the ANOVA shows how the age variable affects *Are letter and form similar*? ability (*F* = 6.59; *P* < 0.05). The descriptive analyses for all variables at Time 1 are reported in Table 2.

**TABLE 2:** Descriptive statistics at Time 1.

**Variables**	**Age**	**Min**	**Max**	***M***	***SD***
Blind touch	4	1.00	5.00	4.24	1.05
	5	0.00	5.00	4.31	1.04
	Total	0.00	5.00	4.27	1.03
Are letters and forms similar?	4	0.00	5.00	2.91	1.68
	5	0.00	5.00	3.89	1.51
	Total	0.00	5.00	3.41	1.65
Snail’s walk	4	0.00	4.00	2.32	1.51
	5	0.00	4.00	2.28	1.70
	Total	0.00	4.00	2.30	1.60

*Min, minimum; Max, maximum; M, media; SD, standard deviation.*

During the Time 2, in reference to the reading ability, the MANOVA emphasizes the main effect linked to the age variable (Wilks’s lambda = 0.65; *F* = 6.62; *P* < 0.01), to the *Snail’s walk* test (Wilks’s lambda = 0.61; *F* = 2.18; *P* < 0.05), and an effect of Blind touch * Letter and form similar interaction (Wilks’s lambda = 0.70; *F* = 2.38; *P* < 0.05). The break down of the univariate effects shows: differences with respect to the age variable (*F* = 4.83; *P* < 0.05) and *Snail’s walk* (*F* = 3.30; *P* < 0.05) in the reading fluency, and the presence of differences with respect to the interaction between *Blind touch* and *Letter and form similar* tests in the reading comprehension (*F* = 4.51; *P* < 0.05). In particular, descriptive analyses show that the older children (7 years old) seem to manifest higher scores in the reading fluency than the younger children (6 years old); children with higher scores at the *Snail’s walk* test seem to present elevated ability in the reading fluency. Furthermore, subjects with lower scores at the *Blind touch* and *Letter and form similar* tests manifest a lower score in the reading comprehension ability. The descriptive analyses for all variables in Time 2 are reported in Table 3.

**TABLE 3:** Descriptive statistics at Time 2.

**Variables**	**Age**	**Min**	**Max**	**M**	**SD**
Blind Touch	6	2.00	5.00	4.29	0.87
	7	2.00	5.00	4.53	0.81
	Total	2.00	5.00	4.41	0.84
Are Letters and Forms Similar?	6	3.00	5.00	4.56	0.61
	7	2.00	5.00	4.81	0.62
	Total	2.00	5.00	4.69	0.63
Snail’s walk	6	1.00	4.00	2.76	0.85
	7	0.00	4.00	2.81	1.21
	Total	0.00	4.00	2.79	1.05
Reading: understanding	6	3.00	5.00	4.56	0.61
	7	2.00	5.00	4.81	0.62
	Total	2.00	5.00	4.69	0.63
Reading: correctness	6	0.00	4.00	2.32	1.51
	7	0.00	4.00	2.28	1.70
	Total	0.00	4.00	2.30	1.60
Reading: fluency	6	1.00	4.00	2.76	0.85
	7	0.00	4.00	2.81	1.21
	Total	0.00	4.00	2.79	1.05
Writing: dictation	6	3.00	15.00	8.68	3.02
	7	4.00	10.00	8.47	1.38
	Total	3.00	15.00	8.57	2.31
Writing: narration	6	1	12	5.50	2.91
	7	0	12	2.75	2.34
	Total	0	12	4.09	2.95
Calculation: written operations	6	0	1	0.71	0.46
	7	0	1	0.94	0.23
	Total	0	1	0.83	0.38
Calculation: written knowledge	6	1	20	6.91	4.24
	7	0	15	3.39	3.13
	Total	0	20	5.10	4.09
Calculation: accuracy	6	0	150	38.79	30.14
	7	0	44	13.31	11.21
	Total	0	150	25.69	25.74
Calculation: time	6	0	4	2.26	1.73
	7	1	4	3.14	0.90
	Total	0	4	2.71	1.43

*The increase of the scores in dictation, spontaneous writing, calculation accuracy, and time indicates a deterioration of the performance (see section “Materials and Methods”). Min, minimum; Max, maximum; M, media; SD, standard deviation.*

During the Time 2, in reference to the writing ability, the second MANOVA emphasizes a main effect linked to the age variable (Wilks’s lambda = 0.78; *F* = 5.36; *P* < 0.01) and to the *Blind touch* ability (Wilks’s lambda = 0.61; *F* = 3.44; *P* < 0.01). The break down of the univariate effects shows differences with respect to: the age variable in the writing dictation (*F* = 8.37; *P* < 0.01) and narration (*F* = 10.54; *P* < 0.01), and the presence of differences with respect to the *Blind touch* test in the writing dictation (*F* = 2.92; *P* < 0.05). In particular, the descriptive analysis shows that the older children (7 years old) seem to manifest fewer mistakes in the writing dictation and narration than the younger children (6 years old). Furthermore, children with a lower score at the *Blind touch* test manifest the higher score in the writing dictation ability.

Finally, in reference to the calculation ability, the MANOVA emphasizes a main effect linked to the age variable (Wilks’s lambda = 0.26; *F* = 25.45; *P* < 0.001) and effect of *Blind touch* * *Snail’s walk* interaction (Wilks’s lambda = 0.45; *F* = 2.03; *P* < 0.05). The breakdown of the univariate effects shows: differences with respect to the age variable in calculation numerical knowledge (*F* = 29.42; *P* < 0.001) and calculation time (*F* = 41.20; *P* < 0.001); furthermore, the breakdown of the univariate effects shows the presence of differences with respect the interaction between *Blind touch* and *Snail’s walk* tests in the calculation time (*F* = 5.14; *P* < 0.01). The descriptive analyses underline that the older children (7 years old) have better performance in the calculation numerical knowledge, although a higher score in the time taken to solve the task than the younger children (6 years old). Furthermore, children with lower scores in *Blind touch* and *Snail’s walk* tests seem to show worst performance in the calculation time ability.

The partial correlation is carried out to verify the role of independent variables (that is age and visual imagery processes) in order to value their effect on dependent variables (that is academic abilities) by eliminating the age variable. In particular, in the first phase a bivariate correlation analysis is performed in order to verify the possible presence of correlations between visual imagery processes, age (independent variables) and scholastic skills (dependent variables). In the second phase a partial correlation analysis is applied among those variables whose correlation was statistically significant, in order to verify the presence of a correlation after eliminating the influence of age variable.

The first analysis underlines the presence of a correlation between *Letters and forms similar* with the spontaneous writing ability (*r* = −0.25; *P* < 0.05); eliminating the age variable - through the partial correlation analysis - the relation does not appear to be significant (*r* = −0.18; *P* = ns). Similarly, the bivariate correlation between *Letters and forms similar* with calculation accuracy (*r* = −0.26; *P* < 0.05) and with written calculation abilities (*r* = 0.21; *p* < 0.05) are significant; eliminating the age variable, both correlations are not significant (*P* = ns).

Therefore, it is not possible to confirm the presence of a real correlation between *Letters and forms similar* with spontaneous writing ability, with calculation accuracy and with written calculation abilities, as the significance is caused by the combination of the relationship between these dimensions with the age variable.

By contrast, the bivariate correlation between *Blind touch* and the writing dictation ability is significant (*r* = −0.38; *P* < 0.01); eliminating the age variable, the correlation still appears significant (*r* = −0.36; *P* < 0.01). Instead the correlation between *Blind touch* and the spontaneous writing appears to be significant (*r* = −0.20; *P* < 0.05), but eliminated age, the correlation does not significant (*r* = −0.16; *P* = ns).

Furthermore, the bivariate correlation between *Snail’s walk* and the calculation accuracy ability appears to be significant (*r* = −0.27; *P* < 0.05); eliminating age, the correlation appears more significant (*r* = −0.31; *P* < 0.01). Similarly, the bivariate correlation between *Snail’s walk* and the calculation speed ability appears to be slightly significant (*r* = −0.24; *P* < 0.05); eliminating age, the correlation appears strongly significant (*r* = −0.31; *P* < 0.01).

Finally, the bivariate correlation between *Snail’s walk* with the writing dictation (*r* = −0.23; *P* < 0.05) and with the spontaneous writing (*r* = −0.23; *P* < 0.05) appear to be significant; eliminating age, both correlations appear more significant (for writing dictation: *r* = −0.24; *P* < 0.05; for spontaneous writing: *r* = 0.25; *P* < 0.05).

Therefore, it is possible to confirm the following relationships: the increase in average scores in *Blind touch* test is related to the increase in writing dictation ability; the increase in the average scores in *Snail’s walk* corresponds to the improvement in calculation accuracy and calculation speed abilities, but also in writing dictation and spontaneous writing abilities.

**FIGURE 1:**
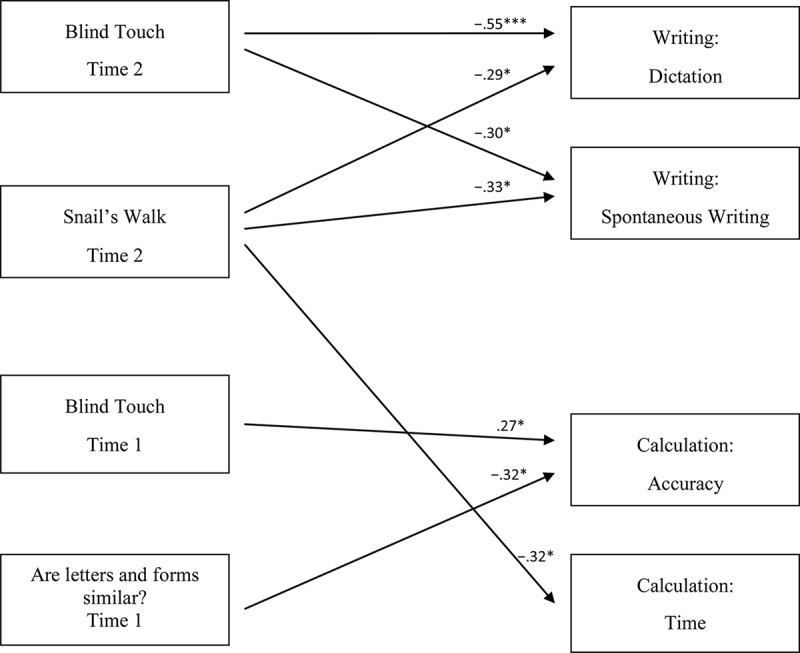
Main effects. ^∗^*p* < 0.05; ^∗∗∗^*p* < 0.001. The increase of the scores in dictation, spontaneous writing, calculation accuracy, and time indicates a deterioration of the performance (see section “Materials”).

Separate hierarchical regression analyses for each dependent variable were conducted to evaluate the contribution of the different mental imagery processes on school learning during different developmental. Into the regression analysis, the variables entered at three hierarchical steps: in order to control possible confounding effects, gender was included in the first step; in order to detect main effects on school learning, in the second and third steps, imagery variables—at Time 1 (children aged 4–5) and Time 2 (children aged 6–7), respectively—were included. The significance of change in squared multiple correlations was assessed at each step. Table 4 underlines the results of the regression models. Figure 1 shows the main effects model.

**TABLE 4:** Hierarchical regressions of imagery ability at Time 1 and Time 2 on school learning.

**Variables**	**β**	***t***	***R*^2^**	**Δ*R*^2^**	***ΔF***
**Reading: understanding**					
*Step 1: Control variable*					
Gender	0.173	1.446	0.030	0.030	2.090
*Step 2: Main variables at Time 1*					
Blind touch	–0.123	–0.911	0.058	0.028	0.645
Are letters and forms similar?	0.074	0.545			
Snail’s walk	–0.109	–0.837			
*Step 3: Main variables at Time 2*					
Blind touch	0.256	1.628	0.123	0.065	1.537
Are letters and forms similar?	–0.014	–0.083			
Snail’s walk	0.178	1.156			
**Reading: correctness**					
*Step 1: Control variable*					
Gender	–0.099	–0.823	0.010	0.010	0.677
*Step 2: Main variables at Time 1*					
Blind touch	0.103	0.771	0.078	0.068	1.610
Are letters and forms similar?	–0.191	–1.416			
Snail’s walk	0.223	1.739			
*Step 3: Main variables at Time 2*					
Blind touch	–0.253	–1.616	0.130	0.051	1.217
Are letters and forms similar?	0.135	0.813			
Snail’s walk	–0.107	–0.697			
**Reading: fluency**					
*Step 1: Control variable*					
Gender	0.102	0.845	0.010	0.010	0.714
*Step 2: Main variables at Time 1*					
Blind touch	0.011	0.077	0.036	0.026	0.584
Are letters and forms similar?	0.136	0.985			
Snail’s walk	–0.138	–1.053			
*Step 3: Main variables at Time 2*					
Blind touch	0.044	0.271	0.07	0.034	0.757
Are letters and forms similar?	0.077	0.447			
Snail’s walk	0.196	1.235			
**Writing: dictation**					
*Step 1: Control variable*					
Gender	0.009	0.076	0.000	0.000	0.006
*Step 2: Main variables at Time 1*					
Blind touch	–0.030	–0.222	0.022	0.022	0.482
Are letters and forms similar?	–0.126	–0.904			
Snail’s walk	0.117	0.882			
*Step 3: Main variables at Time 2*					
Blind touch	–0.546	–3.827^∗∗∗^	0.277	0.255	7.282^∗∗∗^
Are letters and forms similar?	0.012	0.081			
Snail’s walk	–0.292	−2.093^∗^			
**Writing: spontaneous writing**					
*Step 1: Control variable*					
Gender	–0.134	–1.112	0.018	0.018	1.237
*Step 2: Main variables at Time 1*					
Blind touch	0.08	0.598	0.069	0.051	1.182
Are letters and forms similar?	–0.241	–1.777			
Snail’s walk	0.122	0.946			
*Step 3: Main variables at Time 2*					
Blind touch	–0.302	−2.070^∗^	0.244	0.176	4.805^∗∗^
Are letters and forms similar?	–0.165	–1.068			
Snail’s walk	–0.334	−2.338^∗^			
**Calculation: written operations**					
*Step 1: Control variable*					
Gender	0.092	0.765	0.009	0.009	0.586
*Step 2: Main variables at Time 1*					
Blind touch	–0.162	–1.220	0.08	0.071	1.677
Are letters and forms similar?	0.253	1.875			
Snail’s walk	0.100	0.778			
*Step 3: Main variables at Time 2*					
Blind touch	0.103	0.652	0.116	0.036	0.849
Are letters and forms similar?	0.101	0.605			
Snail’s walk	0.161	1.045			
**Calculation: numerical knowledge**					
*Step 1: Control variable*					
Gender	0.028	0.230	0.001	0.001	0.053
*Step 2: Main variables at Time 1*					
Blind touch	–0.207	–1.545	0.068	0.068	1.572
Are letters and forms similar?	0.262	1.932			
Snail’s walk	0.013	0.101			
*Step 3: Main variables at Time 2*					
Blind touch	0.115	0.720	0.090	0.022	0.492
Are letters and forms similar?	0.084	0.496			
Snail’s walk	0.09	0.577			
**Calculation: accuracy**					
*Step 1: Control variable*					
Gender	0.022	0.185	0.001	0.001	0.034
*Step 2: Main variables at Time 1*					
Blind touch	0.270	2.055^∗^	0.103	0.103	2.484
Are letters and forms similar?	–0.316	−2.374^∗^			
Snail’s walk	0.008	0.065			
*Step 3: Main variables at Time 2*					
Blind touch	–0.174	–1.172	0.217	0.113	2.992^∗^
Are letters and forms similar?	–0.220	–1.394			
Snail’s walk	–0.261	–1.796			
**Calculation: time**					
*Step 1: Control variable*					
Gender	0.059	0.486	0.003	0.003	0.236
*Step 2: Main variables at Time 1*					
Blind touch	0.117	0.852	0.026	0.022	0.492
Are letters and forms similar?	0.059	0.426			
Snail’s walk	–0.106	–0.804			
*Step 3: Main variables at Time 2*					
Blind touch	–0.041	–0.254	0.095	0.07	1.594
Are letters and forms similar?	–0.009	–0.055			
Snail’s walk	–0.322	−2.063^∗^			

*^∗^p < 0.05; ^∗∗^p < 0.01; ^∗∗∗^p < 0.001. The increase of the scores in dictation, spontaneous writing, calculation accuracy, and Time indicates a deterioration of the performance (see section “Materials”). β, beta standardized coefficients; *R*^2^, multiple correlation squared - measure of strength of association; *F*, F distribution - Fisher’s F ratio.*

Table 4 illustrates that, in the first step, the introduction of gender as a control variable accounted for a non-significant proportion of the explained variance. This data underlines that controlling for gender does not seem to modify the relations between the variables. Furthermore, gender variable does not seem to be a predictive variable of school learning.

With regard to the main effects at Time 1, both *Blind touch* and *Are letters and forms similar?* predicted calculation accuracy scores (β = 0.27, *p* < 0.05; β = −0.32, *p* < 0.05). As for the main effects at Time 2, the introduction of main variables at Step 3 accounted for an additional proportion of the variance for both writing dictation (Δ*R*^2^ = 0.26, Δ*F* = 7.28, *p* < 0.001) and spontaneous writing (Δ*R*^2^ = 0.18, Δ*F* = 4.81, *p* < 0.01). In particular, both *Blind touch* and *Snail’s walk* predicted writing dictation (β = −0.55, *p* < 0.001; β = −0.29, *p* < 0.05) and spontaneous writing scores (β = −0.30, *p* < 0.05; β = −0.33, *p* < 0.05). *Snail’s walk* predicted calculation speed (β = −0.32, *p* < 0.05).

## Discussion

The research underlined different interesting elements regarding relationships between mental imagery processes and school learning, in different development stages.

Regarding to the capacity to generate a multisensory image at Time 1 (aged 4–5 years), regression analyses show a link between the performance on the *Blind touch* task for children aged 4–5 years and the accuracy of calculation. However, the results of this analysis, which underlined as the children who performed better on this task at Time 1 were less accurate in calculation during the first years of primary school, is of interest and requires further study. Presumably, the simple ability to generate mental images becomes less important for calculation when the early calculation skills are acquired, for, in this stage of calculation learning, the contribution of mental images is mediated by the other skills that are implicated in math performance. In this regard, it is well known that during academic curriculum, arithmetic skills are related to working memory, attention, and phonological decoding (Furst and Hitch, 2000; Fuchs et al., 2006; Commodari and Di Blasi, 2014; Guarnera and D’Amico, 2014).

Regarding to the capacity to create and inspect a mental image, regression analyses show that the performance on the *Are letters and forms similar?* task at Time 1 (aged 4–5) is predictive of the calculation accuracy. In particular, children who obtained high scores on this mental imagery task at Time 1 made fewer calculation errors 2 years later. The results of the study highlight the link between mental imagery processes and math skills, and in particular the connection between the capacity to use mental representations from a preschool age and the ability to compute during the early stages of math learning, confirming the recent literature (Rasmussen and Bisanz, 2005; Barnes et al., 2011; Guarnera et al., 2017).

An interesting aspect of the present results is that *Are letters and forms similar?* task was not predictive the reading and writing skills. The absence of a relationship between this mental imagery task and reading skills depends, presumably, on characteristics of the task. This task measures very basic mental imagery skills, that are a key aspect of developing the prerequisite of reading skills. In preschool, children have, in fact, to acquire the ability to differentiate different shapes and characteristics, such as those that constitute letters. During their first years, children can easily recognize letters; for this reason, the ability measured by the tasks used in this study becomes less important for the future development of academic skills than other skills, such as phonological skills (Vellutino and Scanlon, 1982; Vellutino et al., 2004; Pellerone et al., 2018).

Regarding to the capacity to generate a multisensory image at Time 2 (aged 6–7), regression analyses underline that the *Blind touch* task was predictive of writing skills, namely writing dictation and spontaneous writing abilities. In particular, children who obtained high scores on this mental imagery task made few mistakes on the two writing tasks. These results are consistent with the literature, which has underlined the usefulness of multisensory learning in reading and writing learning (Hulme, 1981; Heller, 1982; Hulme and Bradley, 1984; Hulme et al., 1987; Itakura and Imamizu, 1994; Bara et al., 2004). For example, Hulme et al. (1984), in groups of children aged 3–8 years, show that is possible to achieve a greater level of learning of either real letter names or abstract letter-like forms when asked to find stimuli while producing the name than when they were asked only to repeat the symbol name.

It is interesting to point out that analyses reveal a link between the *Blind touch* task and writing ability but not with reading ability. Researches in this field are not without controversy. Although some studies have highlighted the importance of the tactile component during the acquisition of reading and writing skills (Hulme, 1981; Heller, 1982; Hulme and Bradley, 1984; Hulme et al., 1987; Itakura and Imamizu, 1994; Bara et al., 2004), other studies have not found the same link (Vaughn et al., 1992, 1993; Masterson and Apel, 2006). Masterson and Apel (2006), in order to measure spelling knowledge for keyboard and handwriting tests, presented a list of 40 words to children in Grades 2 through 6. The results of the study indicated that children may spell equally well with a keyboard and a pencil and that spelling knowledge draws on modality-free, lexical representations, which are included in long-term memory.

Regarding the capacity to generate and transform a mental image at Time 2, regression analyses underline that the performance on the *Snail’s walk* task is a predictor of speed calculation. These results enforce the link between calculation abilities and mental imagery, according to the literature (Battista, 1990; Hegarty and Kozhevnikov, 1999; Reuhkala, 2001; McKenzie et al., 2003; Holmes and Adams, 2006). In particular a study conducted by Gunderson et al. (2012) showed that the children’s spatial abilities were predictors of the ability to make approximate calculations when they are eight.

It is interesting to point out that the same task (*Snail’s walk*) at Time 1 does not predict the math abilities. In this regard, it can be hypothesized that the transformation process, which is the most complex in terms of cognitive effort, in the previous stage of development (4–5 years old) is not sufficiently developed to emerge as a predictor of mathematical skills, although it will emerge in the subsequent development phase (6–7 years old).

Furthermore, the *Snail’s walk* task at Time 2 (6–7 years) is predictive of writing skills, namely dictation and spontaneous writing skills. Children who obtained high scores on this mental imagery tasks made few mistakes on the two writing tasks. Even in this case, the results confirm those of previous studies showing that writing also involves visuospatial processes (Hayes and Flower, 1980; Le Bigot et al., 2009).

It is interesting to point out that the data did not show any link between the performances in *Snail’s walk* task and reading skills. Nevertheless, what is the connection between the capacity to transform mental images and linguistic and phonological skills remains ambiguous; in fact, some researches (e.g., Guarnera et al., 2013) did not reveal differences in mental rotation performances between subjects with specific language impairments and those without; instead, other researches discovered that subjects with dyslexia performed worse than their peers without on tasks involving the mental rotation of figures and letters (Gildemeister and Friedman, 1980; Eden et al., 1996; Karádi et al., 2001; Rüsseler et al., 2005).

## Conclusion

In general terms, this study highlights the role of mental imagery processes in school learning. Specifically, the role of generation and inspection processes (basic mental imagery skills that require low cognitive effort) involved in *Blind touch* (generation) and *Are letters and forms similar?* (generation and inspection) tasks emerges at different times of development and as a function of different learning areas. The role of the generation process emerges in the school phase in relation to the learning area of writing, whereas the role of generation and inspection processes emerges only in preschool stage and in relation to the learning area of mathematics. For its part, the role of more complex ability, namely the mental transformation involved in the *Snail’s walk* task, emerges only in the school phase in relation to both writing and mathematics learning.

Therefore the present study shows that the different imagery processes have a different impact on the learning as a function of the development phases and of the different learning areas.

In particular, in reference to the reading ability, it seems that none of the imagery processes considered has a predictive role.

Respect to transformation process, as already mentioned, the literature presents controversial results.

In reference to generation and inspection, it can be assumed that both processes are important in the even earlier stages of learning; in fact, in the first and second years of primary school, the reading acquisition process is already started. Therefore it would be interesting to verify the role of generation and inspection in more basic tasks, such as the recognition and discrimination of letters and syllables, rather than in reading passages.

Relative to writing, it seems that during 6–7 years old, generation and transformation processes are important. It can be hypothesized that - unlike reading - writing requires the support of imagery processes even in a learning acquisition phase that is already underway. In writing tasks, in fact, the ease of accessing orthographic representations, intended both as generation of individual letters and as transformation of the single sign when this must be combined with other signs that follow it or precede it, to form words, could be useful.

Regarding to the mathematics, it seems that both inspection and transformation processes are important but during different stages of development. The inspection, which implies the ability to grasp the details of the representations (for example both the numbers and the algorithmic symbols differ in small details), which us relevant to 4–5 years, ceases to be relevant at 6–7 years, to leave the place to the process of transformation, which is involved in many aspects of mathematical ability.

In sum, the results of the present study demonstrate that the different aspects in which mental imagery processes are articulated seem to be involved in some skills that constitute school learning. In fact the present research has focused on overtly observable performance effects during early skill acquisition, although neglecting the investigation of covert cognitive effects. From a theoretical point of view, future research might focus on the question how different domain-general ability contribute to the development of visual imagery processes.

The present study contains some limitations which must be acknowledged. The first limitation was the statistical population: in particular the reduced size of participant group, although being a longitudinal study, determines a difficulty to generalize these findings; for this reason, this study needs to be replicated with a larger group.

Another limitation is associated with an instrument restriction; in particular, the assessment of basic levels of development are not provided to allow the samples to be homogenized and to be able to affirm that the differences are in the measured aspects and not in the general development or levels of yield. Because of the large number of tests being administered, to include other instruments - in order to value the general developmental level - was not feasible.

These findings emphasize the potential usefulness of a screening for mental imagery ability for schoolchildren to adopt effective measures to increase their mental imagery abilities. Further studies should investigate the relationship between mental imagery and learning difficulties. In fact, further studies should focus on this link, for mental imagery training could be useful not only for promoting school success but also for preventing learning difficulties. Intensive prevention trials focused on the use of mental imagery are relatively inexpensive and could easily be carried out at schools and managed by teachers.

## Data Availability

All datasets generated for this study are included in the manuscript and/or the supplementary files.

## Ethics Statement

The administered survey was reviewed and approved by the Ethics Commission of Kore University of Enna, Italy.

## Author Contributions

GV and SB performed the test administration and preliminary data analyses. MG and EC participated in conducting the literature review, and drafting the introduction, research objectives, and conclusions. MP made contributions in the study design, carrying out the statistical analyses, and drafting the results and the conclusions.

## Conflict of Interest Statement

The authors declare that the research was conducted in the absence of any commercial or financial relationships that could be construed as a potential conflict of interest.
